# Supplementary material for: The neonatal southern white rhinoceros ovary contains oogonia in germ cell nests
Source: Commun Biol. 2023 Oct 17;6:1049. doi: 10.1038/s42003-023-05256-5 (PMC10582104; doi:10.1038/s42003-023-05256-5)
Supplement: Supplementary file 2 — Reporting Summary [file 42003_2023_5256_MOESM2_ESM.pdf]

nature portfolio

Corresponding author(s): Suzannah Williams

Last updated by author(s): Aug 12th 2023

Reporting Summary

Nature Portfolio wishes to improve the reproducibility of the work that we publish. This form provides structure for consistency and transparency in reporting. For further information on Nature Portfolio policies, see our Editorial Policies and the Editorial Policy Checklist.

Statistics

For all statistical analyses, confirm that the following items are present in the figure legend, table legend, main text, or Methods section.

n/a	Confirmed	
☐	☒	The exact sample size (n) for each experimental group/condition, given as a discrete number and unit of measurement
☐	☒	A statement on whether measurements were taken from distinct samples or whether the same sample was measured repeatedly
☐	☒	The statistical test(s) used AND whether they are one- or two-sided<br>*Only common tests should be described solely by name; describe more complex techniques in the Methods section.*
☒	☐	A description of all covariates tested
☒	☐	A description of any assumptions or corrections, such as tests of normality and adjustment for multiple comparisons
☒	☐	A full description of the statistical parameters including central tendency (e.g. means) or other basic estimates (e.g. regression coefficient) AND variation (e.g. standard deviation) or associated estimates of uncertainty (e.g. confidence intervals)
☒	☐	For null hypothesis testing, the test statistic (e.g. F, t, r) with confidence intervals, effect sizes, degrees of freedom and P value noted<br>*Give P values as exact values whenever suitable.*
☒	☐	For Bayesian analysis, information on the choice of priors and Markov chain Monte Carlo settings
☒	☐	For hierarchical and complex designs, identification of the appropriate level for tests and full reporting of outcomes
☒	☐	Estimates of effect sizes (e.g. Cohen's d, Pearson's r), indicating how they were calculated

Our web collection on statistics for biologists contains articles on many of the points above.

Software and code

Policy information about availability of computer code

Data collection	No software was used
Data analysis	No software was used

For manuscripts utilizing custom algorithms or software that are central to the research but not yet described in published literature, software must be made available to editors and reviewers. We strongly encourage code deposition in a community repository (e.g. GitHub). See the Nature Portfolio guidelines for submitting code & software for further information.

Data

Policy information about availability of data

All manuscripts must include a data availability statement. This statement should provide the following information, where applicable:

- Accession codes, unique identifiers, or web links for publicly available datasets
- A description of any restrictions on data availability
- For clinical datasets or third party data, please ensure that the statement adheres to our policy

The data that support the findings of this study are available from the corresponding author on reasonable request.

Human research participants

Policy information about studies involving human research participants and Sex and Gender in Research.

Reporting on sex and gender	This information has not been collected.
Population characteristics	This information has not been collected.
Recruitment	This information has not been collected.
Ethics oversight	This information has not been collected.

Note that full information on the approval of the study protocol must also be provided in the manuscript.

Field-specific reporting

Please select the one below that is the best fit for your research. If you are not sure, read the appropriate sections before making your selection.

☒ Life sciences ☐ Behavioural & social sciences ☐ Ecological, evolutionary & environmental sciences

For a reference copy of the document with all sections, see nature.com/documents/nr-reporting-summary-flat.pdf

Life sciences study design

All studies must disclose on these points even when the disclosure is negative.

Sample size	No sample size calculation was performed due to the scarcity of the samples. 1 neonatal and 3 adult rhinoceros ovaries were investigated. Due to the low number of follicles that could be investigated, no statistical tests comparing the neonatal or adult individuals or follicle classes could be performed on the follicular parameters measured.
Data exclusions	No data were excluded from the study
Replication	It is a descriptive study on 1 neonatal and 3 adult rhinoceros ovaries. All data observed in all samples were used and described.
Randomization	Randomization is not relevant to this study. All samples were investigated and the observations were all described.
Blinding	When describing the structures in the ovaries, blinding was not relevant.<br>Samples were blinded for the numeric parameters of different follicle stages in the neonatal and adult ovary, samples were blinded during calculation of the different parameters. After all measurements were finalised, it was revealed which sample each follicle belonged to.

Reporting for specific materials, systems and methods

We require information from authors about some types of materials, experimental systems and methods used in many studies. Here, indicate whether each material, system or method listed is relevant to your study. If you are not sure if a list item applies to your research, read the appropriate section before selecting a response.

Materials & experimental systems

n/a	Involved in the study	
☐	☒	Antibodies
☒	☐	Eukaryotic cell lines
☒	☐	Palaeontology and archaeology
☒	☐	Animals and other organisms
☒	☐	Clinical data
☒	☐	Dual use research of concern

Methods

n/a	Involved in the study	
☒	☐	ChIP-seq
☒	☐	Flow cytometry
☒	☐	MRI-based neuroimaging

Antibodies

Antibodies used	The describtion of the antibodies is also included in Table 3 in the manuscript.<br>Primary antibody - Function - Catalogue - Species - Concentration - Secondary antibody - Catalogue - Dilution<br>1) collagen I - extracellular matrix protein - MA1-26771 - mouse monoclonal - 25 µg/ml - goat anti-mouse IgG - BA-9200 - 1:200<br>2) Ki-67 - proliferation marker - PA5-19462 - rabbit polyclonal - 5 µg/ml - goat anti-rabbit IgG - BA-1000 - 1:200

3) minichromosome maintenance complex component 2 (MCM2) - proliferation marker - PA5-79645 - rabbit polyclonal - 2.5 µg/ml - goat anti-rabbit IgG - BA-1000 - 1:200
4) sex determining region Y-box 2 (SOX2) - pluripotency factor - PA1-16968 - rabbit polyclonal - 5 µg/ml - goat anti-rabbit IgG - BA-1000 - 1:200
5) octamer-binding transcription factor 4 (Oct4 or POU5F1) - pluripotency factor - 11263-1-AP - rabbit polyclonal - 3.75 µg/ml - goat anti-rabbit IgG - BA-1000 - 1:200
6) DEAD-Box Helicase 4 (DDX4 or Vasa) - germ cell marker - PA5-23378 - rabbit polyclonal - 5 µg/ml - goat anti-rabbit IgG - BA-1000 - 1:200
7) anti-Müllerian hormone (AMH) - hormone - PA5-35851 - rabbit polyclonal - 5 µg/ml - goat anti-rabbit IgG - BA-1000 - 1:200
8) cannabinoid receptor 1 (CB1) - G protein-coupled receptor - 17978-1-AP - rabbit polyclonal - 5.5 µg/ml - goat anti-rabbit IgG - BA-1000 - 1:200
9) cluster of differentiate 20 (CD20) - B-lymphocyte marker - PA5-16701 - rabbit polyclonal - 0.1925 µg/ml - goat anti-rabbit IgG - BA-1000 - 1:200
10) sodium potassium ATPase (NaKATPase) - membrane marker - ab58475 - rabbit polyclonal - 10 µg/ml - goat anti-rabbit IgG - BA-1000 - 1:200

Validation

Since none of the antibodies was validated by the manufacturer for rhinoceros tissue, we validated the antibodies ourselves. We always included a negative control in the immunohistochemical experiments and included a mouse ovary as a positive control when this was also identified as cross-reacting to the antibody. The negative controls (included in all figures) demonstrated that the observed signals were specific. When the sample did not show a positive signal in the investigated structure, a structure in the sample, known to have to be positive, was used as an internal positive sample control.
